# Short-term rainfall forecast model based on the improved BP–NN algorithm

**DOI:** 10.1038/s41598-019-56452-5

**Published:** 2019-12-24

**Authors:** Yang Liu, Qingzhi Zhao, Wanqiang Yao, Xiongwei Ma, Yibin Yao, Lilong Liu

**Affiliations:** 1 College of Geomatics, Xi’an University of Science and Technology, Xi’an, 710054 China; 2 School of Geodesy and Geomatics, Wuhan University, Wuhan, 430072 China; 3 College of Geomatics and Geoinformation, Guilin University of Technology, Guilin, 541004 China

**Keywords:** Hydrology, Natural hazards

## Abstract

The existing methods have been used the Zenith Total Delay (ZTD) or Precipitable Water Vapor (PWV) derived from Global Navigation Satellite System (GNSS) for rainfall forecasting. However, the occurrence of rainfall is highly related to a myriad of atmospheric parameters, and a good forecast result cannot be obtained if it only depends on a single predictor. This study focused on rainfall forecasting by using a number of atmospheric parameters (such as: temperature, relative humidity, dew temperature, pressure, and PWV) based on the improved Back Propagation Neural Network (BP–NN) algorithm. Results of correlation analysis showed that each meteorological parameter contributed to rainfall. Therefore, a short-term rainfall forecast model was proposed based on an improved BP–NN algorithm by using multiple meteorological parameters. Two GNSS stations and collocated weather stations in Singapore were used to validate the proposed rainfall forecast model by using three years of data (2010–2012). True forecast (TFR), false forecast (FFR), and missed forecast (MFR) rate were introduced as evaluation indices. The experimental result revealed that the proposed model exhibited good performance with TFR larger than 96% and FFR of approximately 40%. The proposed method improved TFR by approximately 10%, whereas FFR was comparable to existing literature. This forecasted result further verified the reliability and practicability of the proposed rainfall forecasting method by using the improved BP–NN algorithm.

## Introduction

Water vapor is the most important and abundant greenhouse gas in the troposphere and plays an important role in atmospheric radiation, energy balance, and hydrological cycle^1,2^. However, accurately monitoring this greenhouse gas in the troposphere is difficult because of its low content, extremely uneven distribution, and rapid changes^3,4^. Precipitable water vapor (PWV) refers to the amount of precipitation formed by the condensation of water vapor into rain in the air column of the unit cross section from the ground to the top of the atmosphere; it can be used to quantify the content of water vapor in the troposphere^5^. The accurate detection of PWV provides the basis for numerical weather prediction^3–6^.

At present, the conventional methods of PWV detection mainly include radiosonde and water vapor radiometer (WVR). Radiosonde can provide water vapor products with high vertical resolution, and the vertical resolution of radiosonde data can be as high as 30 m^7,8^, or even 5 m^9^. However, the spatial–temporal resolutions of the PWV data obtained by this method are low because the distance between the adjacent stations is 200–300 km and the sounding balloon is launched only two to four times a day^10,11^. Such spatial–temporal resolutions can not satisfy the requirements of small- medium scale atmospheric water vapor change and weather prediction. WVR can provide water vapor products with high temporal resolution, but it has not been widely used because of its expensive equipment and vulnerability to cloud and rainfall^10–13^. Although satellite image products can provide precipitation information with high spatial resolution, these methods are used rarely due to their low accuracy^11,14^.

The Global Navigation Satellite System (GNSS) can be used in remote sensing of atmospheric water vapor given its continuous development and progress. Askne and Nordius^15^ deduced the functional relationship between Zenith Wet Delay (ZWD) and PWV via experimentation and proposed the method of detecting atmospheric water vapor by using ground-based GNSS technology. Bevis *et al*.^16^ first used GNSS observation to estimate PWV that promoted the development of GNSS meteorology. The retrieval of PWV by using GNSS technology is widely used in meteorology because of its high spatial and temporal resolutions (1 second to 2 hours, several kilometers to tens of kilometers), all-weather conditions, high accuracy (<2 mm), and low cost^1,10^.

Recent studies have used GNSS-derived zenith total delay (ZTD) or PWV to forecast rainfall^17^. found that the PWV value is sharply increased before the abrupt rainfall events^18^. proved that PWV is a good indicators and can help to improve the physics of a weather model. Benevides *et al*.^3^ proposed a simple rainfall prediction model by fitting PWV time series data via the least squares method. The true forecast rate of the model was 75%, and the false forecast rates were between 60% and 70% in Lisbon, Portugal. Yao *et al*.^4^ also built a rainfall prediction model by using the PWV data of five GNSS stations in Zhejiang Province, and the true and the false forecast rate of the rainfall forecast model were approximately 80% and 66%, respectively. Zhao *et al*.^6^ proposed a rainfall forecast algorithm by using PWV in its ascending period and applied this method to the prediction of typhoon events. The true forecast rate was approximately 70%, but the false forecast rate was only 18%. Manandhar *et al*.^5^ built a rainfall forecast model by using 30 min of PWV time series data to predict the rainfall in the next 5 minutes, and the true forecast rate was approximately 87.7%, whereas the false forecast rate was 38.6% in Singapore. During the retrieval of GNSS-derived PWV, errors have been introduced due to the observed error in the meteorological data and the conversion error from ZWD to PWV. To overcome these issues, Zhao *et al*.^19^ proved the feasibility of using ZTD directly to forecast rainfall and proposed a rainfall forecast algorithm using ZTD variation and its first derivative. The true and false forecast rates of this algorithm were 85% and 66%, respectively.

Artificial neural network (ANN) has attracted considerable attention from researchers in the field of artificial intelligence. ANN abstracts the brain as a neural network and establishes a simple model connecting different networks during information processing^20,21^. Back propagation (BP)–NN is a kind of multilayer feed forward artificial neural network with mono directional transmissions^22,23^, which has the advantages of memory association, solving complex internal mechanism problems, independent learning and adaptive ability, and parallel processing of data^24^. In addition, neural networks can extract the input–output relationship without explicit physical conditions^25^ and make use of error gradient descent algorithm to minimize the mean square error between the output value of network and the actual output value^26^. Therefore, neural networks are suitable for meteorological prediction research. Guan *et al*.^20^ proved that the BP algorithm can be applied to high-precision rainfall prediction by using the precipitation data of 26 base stations in the Chaohe River basin from 1958 to 2012. Hashim *et al*.^27^ also found that the BP neural network is suitable for the study of rainfall prediction with meteorological parameters, such as temperature, air pressure, and humidity. Srivastava *et al*.^28^ predicted the daily rainfall in northern India by using the ANN algorithm and achieved good forecasted results. Manandhar *et al*.^29^ successfully used the machine learning algorithm called support vector machine (SVM) to classify precipitation and nonprecipitation events. The advantage of neural network is that they are best suited to solving the problems that are the most difficult to solve by traditional computational methods^30^, Neural networks can learn from examples (past data) recognize a hidden pattern in historical observations and use them to forecast future values^31^. In addition^32^, proposed a multilayer feedforward neural network (the NN) model for weighted mean temperature of atmospheric water vapor predicting, and the result shows the good performance of NN model on global scale^33^. proposed a new ZTD model based on a back propagation neural network, and the ZTD prediction accuracy has been improved by more than 12.4%.

At present, some algorithms have been used to forecast rainfall by using the GNSS-derived ZTD or PWV to obtain good forecasting results. However, the incidence of false alarm in these studies is high (60%–70%), and the true forecasted rate is unstable in different experiments (70–90%) because the occurrence of rainfall is highly correlated with considerable atmospheric parameters. Moreover, this type of prediction process cannot be described accurately by only a single predictor (PWV or ZTD). These studies mentioned above provided a new idea on how to forecast rainfall from the following aspects: (1) using an increased number of meteorological parameters to describe the occurrence of rainfall as much as possible and (2) introducing the neural network algorithm to forecast rainfall that becomes the focus of this study. A rainfall forecast model was proposed by using the improved BP–NN algorithm with multiple meteorological parameters (PWV; temperature, T; relative humidity, RH; dew point, DPT; day of year, DoY; hour of day, HoD; and pressure, P). The numerical experiment revealed that this method can forecast the possibility of rainfall in a short amount of time (10–60 minutes), and good performance was obtained by the proposed rainfall forecast method.

## GNSS-Derived PWV and Theory of BP-NN Algorithm

### Retrieval of GNSS-derived PWV

ZTD occurs as the GNSS signal is affected by the atmospheric refraction when it passes through the troposphere, ZTD includes zenith hydrostatic delay (ZHD) and ZWD^34^.

1 $$ZTD=ZHD+ZWD$$

ZHD accounts for approximately 90% of ZTD and is mainly affected by latitude and surface pressure^35^. ZWD is related to the moisture content in the signal propagation path, and GNSS signals are affected by the polar motion of water vapor molecules^10^. ZHD can be calculated accurately by using the following empirical formula^35^:

2 $$ZHD=\frac{0.0022768\cdot {P}_{W}}{1-0.002266\cdot cos(2\varphi )-0.00028\cdot H}$$

where *P*_*W*_ is the surface pressure of the station with a unit of °C, *φ* refers to the latitude of the station with a unit of radian, and *H* is the geodetic height of the station with a unit of km. Therefore, ZWD can be obtained by extracting ZHD from ZTD, and PWV can be calculated by multiplying the conversion factor as follows^19^:

3 $$PWV=\frac{\varPi \cdot ZWD}{{\rho }_{W}}$$

where *ρ*_*W*_ is the water vapor density, and Π represents the conversion factor, which can be expressed as follows:

4 $$\begin{array}{c}\varPi =[\,-\,1\cdot sgn(\varphi )\cdot 1.7\cdot {10}^{-5}{|\varphi |}^{{H}_{f}}-0.0001]\cdot cos(\frac{DoY-28}{365.25}\cdot 2\pi )\\ \,+[0.165-(1.7\cdot {10}^{-5}){|\varphi |}^{1.65}]+(-2.38\cdot {10}^{-6})\cdot H\end{array}$$

where *H*_*f*_ = 1.48 or *H*_*f*_ = 1.25 when the station is located in the northern or southern hemisphere, respectively; and *DoY* represents the day of the year. Equation (4) is an empirical formula that is fitted by using 174 radiosonde stations over a period of four years in tropical, subtropical, and temperate regions. The accuracy of the retrieved PWV by using this equation is ±1 mm^14^.

### Theory of BP–NN algorithm

**Figure 1:**
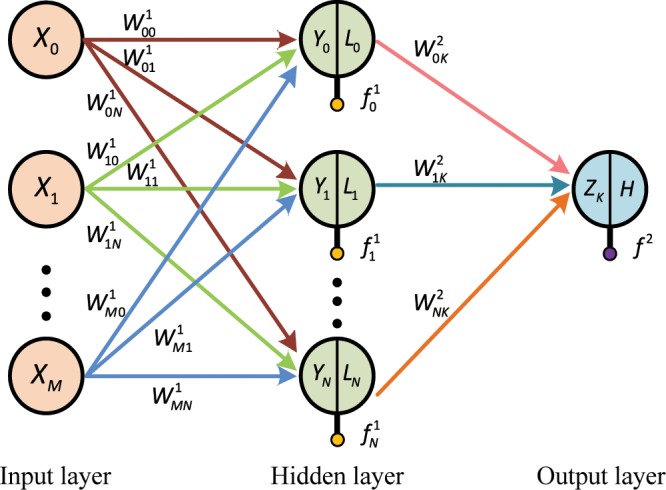
Topological structure of the BP–NN algorithm. [The figure is plotted by VISIO 2010 (https://products.office.com/zh-cn/Visio/flowchart-software.html)].

BP–NN consists of input, hidden, and output layers. Each layer is fully interconnected, and no interconnection exists in the same layer. One or more hidden layers can exist. Robert Hecht–Nielsen^36^ proved that any complex nonlinear problem can be simulated with a three-layer BP–NN algorithm, and any mapping from N- to M-dimensions can be completed. Therefore, this study adopts a three-layer BP–NN structure. Figure 1 shows that the BP–NN structure has input, implicit, and output layers.

The mathematical principle of the forward propagation of BP–NN is as follows^37^:

5 $${Y}_{j}={\varSigma }_{i=0}^{M}{W}_{ij}^{1}{X}_{i}+{f}^{1}M$$

where *X*_*i*_ is the input vector, *M* is the number of input layer nodes and $i\in (0,M)$, ${W}_{ij}^{1}$ is the weighted value between the *i* th neurons in the input layer and the *j* th neurons in the hidden layer, *f* ^1^ is the threshold parameter of the hidden layer, *Y*_*j*_ is the node input value of the hidden layer and $j\in (0,\,N)$, and *N* is the number of hidden layer nodes. The input value of each hidden layer node is converted to the output value *L*_*j*_ of the corresponding hidden layer node through the nonlinear transfer function. The following sigmoid function is a widely used transfer function of the hidden layer^38^:

6 $${L}_{j}=f({Y}_{j})=\frac{1}{1+{e}^{-{Y}_{j}}}$$

The output layer is calculated similar to that of the hidden layer and expressed as follows:

7 $${Z}_{K}={\varSigma }_{j=0}^{N}{W}_{jK}^{2}{L}_{j}+{f}^{2}$$

where ${W}_{jK}^{2}$ is the weighted value between the *j* th unit in the hidden layer and the output layer unit *Z*_*k*_, $j\in (0,\,N)$; *f*  ^2^ is the threshold parameter of the output layer; *Z*_*k*_ is the input value of the output layer node; and the following linear function ReLU is a widely used transfer function of the output layer^27^:

8 $$H=f({Z}_{K})=max(0,{Z}_{K})$$

where *H* is the output value of the output layer node.

The above equation is the forward propagation mode of the BP–NN algorithm. The input information is transmitted from the input layer to the output layer through the hidden layer. If the output results do not match the expectations, then they enter the following reverse propagation process: the error starts from the output layer, passes through the hidden layer, and finally reaches the input layer, thereby completing a reverse propagation. In the BP process, the weights of each layer are corrected by decreasing the error gradient. The weights between the *i* th neuron in the input layer and the *j* th neuron in the hidden layer are corrected as follows^27^:

9 $${W}_{ji}(t)={W}_{ji}(t-1)+{\eta }_{a}{\rho }_{j}(t){x}_{i}(t)+{\alpha }_{a}\varDelta {W}_{ji}(t)$$

10 $${f}_{j}(t)={f}_{j}(t)+{\eta }_{b}{\rho }_{j}(t){x}_{i}(t)+{\alpha }_{b}\varDelta {f}_{j}(t)$$

where *W* and *f* are the weight value and threshold, respectively; *α*_*a*_ and *α*_*b*_ are the momentum constants used to determine the effect of the last step parameter change on the current propagation direction; ${\eta }_{a}$ and ${\eta }_{b}$ refer to the learning rates; ${\rho }_{j}(t)$ is the *j* th neuron error signal of the hidden layer in the process of BP–NN algorithm. The output layer neuron error signal $\rho (t)$ can be expressed as follows^39^:

11 $$\rho (t)=\frac{1}{2}{\sum }_{p=1}^{G}{[H-\hat{H}]}^{2}$$

where *G* is the number of data in the training data set, *H* is the desired output, and $\hat{H}$ is the actual output. The process of forward and backward propagations is repeated until the error between the output and the expectation is reduced to an acceptable level or the number of learning times reaches a predetermined value.

## Data and Experiment Description

### Data description

**Figure 2:**
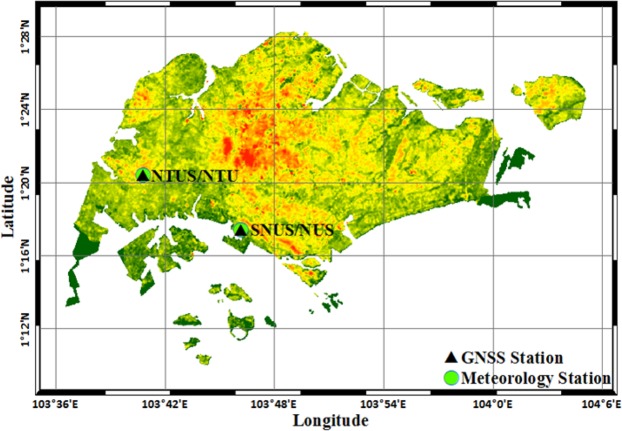
Geographic distribution of the ground-based GNSS stations and the collocated meteorological stations in Singapore. [The figure is plotted by MATLAB 2016a (https://cn.mathworks.com/products/matlab.html)].

Two GNSS stations and the collocated meteorological stations in Singapore were selected over the period of 2010 to 2012 to perform the experiment. Figure 2 presents the geographic distribution of the selected GNSS stations. One of the GNSS stations, NTUS, belongs to the International GNSS Service (IGS). Another station SNUS belongs to the Singapore Satellite Positioning Reference Network (SiReNT) and located in the National Technological University. GNSS observations of NTUS station was downloaded from ftp://cddis.gsfc.nasa.gov/pub/gps/data/. GIPSY OASIS II was used to process the GNSS observations to obtain the ZTD parameters^40^. The Global Mapping Function (GMF) is used and the elevation cut-off angle of 10° is selected for GNSS observations. ZWD data were calculated based on Eqs. (1) and (2). Finally, the PWV data with the intervals of 5 minutes were obtained based on Eqs. (3) and (4). Here, the PWV data of SNUS station is replaced by that of NTUS station. This because that (1) the distance between two stations is very close (about 11 km) and (2) the GNSS observations from SiReNT cannot be obtained currently.

The collocated meteorological data were also obtained from meteorological stations NUS and NTU, and Table 1 lists the corresponding information of the meteorological stations. In station NUS, seven meteorological parameters were collected, including surface pressure (P), surface temperature (T), DoY, hour of day (HoD), minute of hour (MoH), RH, and rainfall with the time resolution of 5 minutes. In station NTU, T, RH, DPT, DoY, HoD, MoH, and rainfall are collected with the time resolution of 1 minute. To unify the time resolution of meteorological parameters and GNSS-derived PWV data, the meteorological parameters in NTU were resampled every 5 minutes.

**Table 1:** Detailed information of each GNSS meteorological station used in the experiment.

GNSS/Meteorological station	Longitude (°)	Latitude (°)	Height (m)	Time resolution	Periods
NTUS/NTU	103.68	1.34	78	5 min	2010–2012
SNUS/NUS	103.77	1.29	63	5 min	2010–2012

### Improved BP–NN algorithm and the selection of key parameters

An improved weight correction method of BP–NN algorithm was proposed by using the Levenberg–Marquardt (L–M) learning rules to overcome the disadvantages of slow convergence speed, local minimum, and training paralysis of the traditional BP neural network. The L–M formula is presented as follows:

12 $$\varDelta W={({J}^{T}J+\mu I)}^{-1}{J}^{T}e$$

where Δ*W* is the corrected weight by using the L–M method, *J* is the Jacobian matrix of the network error to the weight derivative, *e* is the error vector, and *μ* is a scalar. When *μ* = 0, the Newton method is used in the L–M equation, whereas the gradient method is used when *μ* is a large value. Compared with the traditional BP neural network learning method, the improved correction method has the following advantages: (1) rapid convergence rate, (2) ability to combine the advantages of gradient descent and Newton methods, and (3) performance stability^27^.

Two important parameters must be set for the BP–NN algorithm, namely, the number of hidden layer nodes and learning rate. Therefore, selecting an appropriate method in determining these parameters is crucial to establishing the rainfall forecast model by using the BP–NN algorithm. If the number of hidden layer nodes is extremely small, the convergence speed of the whole neural network will slow down and it is difficult to conduct, and the trained result of the BP–NN algorithm cannot be obtained or the algorithm cannot recognize the samples that were previously unavailable and the fault tolerance is poor; if the number of hidden layer nodes is extremely large, then the learning time is increased and the generalization ability of the BP–NN algorithm is reduced^41,42^. The number of hidden layer nodes is selected according to Kolmogrov’s theorem. An equal relationship exists between the number of input layer neurons and the number of hidden layer neurons^23,43^, and the calculation of which is presented as follows:

13 $${N}_{hid}=2\times {N}_{in}+1$$

where *N*_*hid*_ and *N*_*in*_ are the number of hidden and input layer nodes, respectively. According to Kolmogrov’s theorem, the number of selected hidden layer nodes can express any mapping accurately and coordinate the capacity and training time of the hidden layer^23,43^. The selection of learning rate has attracted the interest of many scholars in the research of BP–NN. If the learning rate is extremely small, then the convergence of the neural network can be guaranteed. However, the number of iterations required is large, and the convergence speed is slow. If the learning rate is extremely large, then it may be overcorrected, making it difficult to perform convergence of the neural network^26^. The learning rate is selected based on the following the empirical formula proposed by Kung and Hwang^44^:

14 $$\eta =2/({N}_{hid}+1)$$

where *η* and *N*_*hid*_ are the learning rate and the number of hidden layer nodes, respectively.

### BP–NN experiment

Three schemes are designed for the two selected stations by using the improved BP–NN algorithm. Each scheme includes the following aspects: BP–NN (1) simulated and (2) forecasted experiments. With Scheme 1 in the SNUS station as an example, the BP–NN simulation experiment is carried out first by using the meteorological data of 2010 to obtain the rainfall forecast model of 2010 by using the BP–NN algorithm. Then, the meteorological data of 2010 in the SNUS station are input into the rainfall forecast model to obtain the rainfall simulation results of 2010. Finally, the meteorological data of 2011 in the SNUS station are input into the 2010 rainfall forecast model to obtain the forecasted results of 2011. Table 2 presents the experiment information and schemes designed in two stations.

**Table 2:** Specific information on the experiment performed based on the improved BP–NN algorithm.

Station	SNUS		NTUS	
Experiment	Simulated period	Forecasted period	Simulated period	Forecasted period
Scheme 1	2010	2011	2010	2011
Scheme 2	2011	2012	2011	2012
Scheme 3	2010 + 2011	2012	2010 + 2011	2012
Input information	P, T, DoY, HoD, MoH, RH and PWV		T, RH, DPT, DoY, HoD, MoH and PWV	
Output information	Rainfall		Rainfall	

**Figure 3:**
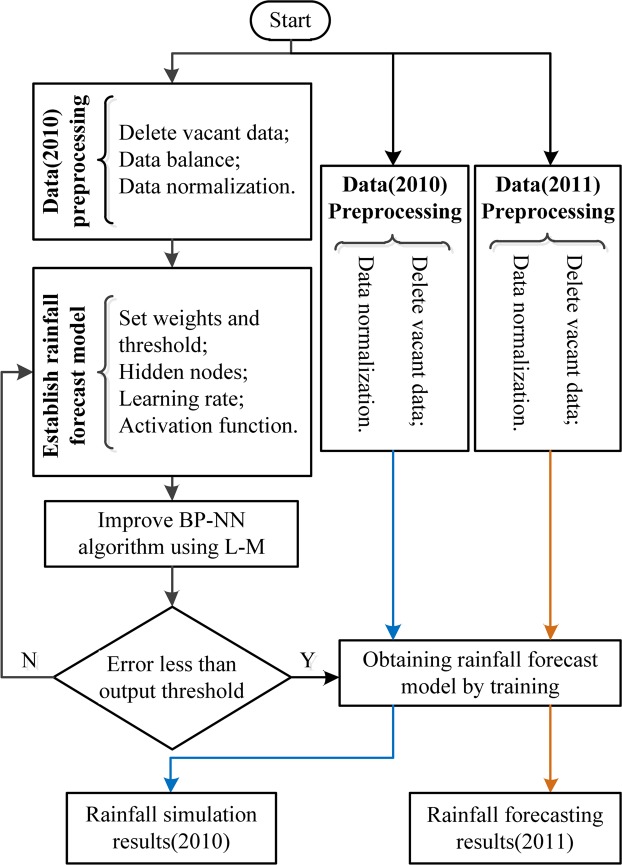
Flowchart of the rainfall forecast model based on the improved BP–NN algorithm. [The figure is plotted by VISIO 2010 (https://products.office.com/zh-cn/Visio/flowchart-software.html)].

Figure 3 shows the flowchart of the BP–NN experiment that includes the technical route of simulated and forecasted experiments. Equalization and normalization processes of the input data are initially performed, and the relevant parameters of the rainfall forecast model with the BP–NN algorithm are then set up. Then, the rainfall forecast model can be established. Finally, the simulated result in 2010 and the forecasted result in 2011 can be obtained by using the established rainfall forecast model. Tests are performed by using the BP–NN algorithm, and the empirical error threshold of 1 × *e*^−5^ is selected between the output and expectation in Eq. (11).

## Rainfall Forecasts Based on the Improved BP–NN Algorithm

### Data and the correlation analysis

Some data are unavailable in some time periods due to the instability of equipment or weather factors. Therefore, the collected meteorological data should be analyzed initially. Table 3 presents the statistical result of the collected meteorological data in SNUS and NTUS stations for three years (2010–2012). Among them, SNUS stations had the most remarkable vacancies in data in 2010 with a vacancy rate of 47.09%, followed by NTUS stations in 2011 with a datum vacancy rate of 33.46%. The datum vacancy rates of SNUS and NTUS stations in 2012 were relatively small at approximately 17%. The datum vacancies of SNUS stations in 2011 and NTUS stations in 2010 were comparable. Prior to the experiment, marking the position and deleting unavailable data are necessary to remove their influence on the prediction accuracy of the BP–NN training model.

**Table 3:** Statistical result of the collected meteorological data in SNUS and NTUS stations for three years (2010–2012).

Data type	SNUS			NTUS		
	2010	2011	2012	2010	2011	2012
Total data (epoch)	105120	105120	105408	105120	105120	105408
Available data (epoch)	55622	92765	87030	92594	69946	87307
Missing data (epoch)	49498	12355	18378	12526	35174	18101
Vacancy rate (%)	47.09	11.75	17.43	11.91	33.46	17.17

**Figure 4:**
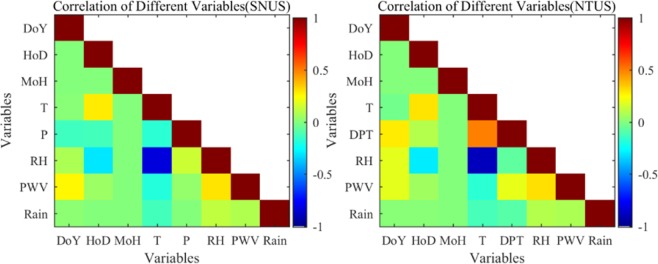
Correlation between different meteorological factors and rainfall at the SNUS and NTUS stations from 2010 to 2012. [The figure is plotted by MATLAB 2016a (https://cn.mathworks.com/products/matlab.html)].

The correlation between different meteorological parameters and rainfall should be analyzed prior to the BP–NN experiment because if a strong correlation exists between the two variables, then the second variable will not contribute additional classification information to the classification process. Therefore, the second variable does not function as a classification factor^45^. Figure 4 shows the correlation between rainfall and each meteorological parameter for two stations from 2010 to 2012.

This figure shows that no strong correlation exists between meteorological parameters and rainfall, thereby indicating that the occurrence of rainfall is related not only to the meteorological parameters in the experiment but also to other meteorological parameters or meteorological processes. The correlation coefficients between T and RH were the largest with values of −0.83 and −0.90 in SNUS and NTUS stations, respectively, thereby indicating that a strong negative correlation exists between the two variables. A positive correlation exists between HoD and T with the correlation coefficients of 0.28 and 0.32 in the two stations, respectively. These results indicated that the temperature changed with the alternation of day and night. A relatively low correlation appeared between rainfall and PWV with a value of approximately 0.1 in the two stations, thereby explaining the high false alarm rate when only PWV/ZTD was used for rainfall forecasting. In addition, a positive correlation between rainfall and other meteorological parameters (DoY, HoD, MoH, RH, and PWV) indicated that rainfall was affected by these parameters to some degree. Therefore, selecting these meteorological parameters as predictors from the perspective of correlation analysis is reasonable.

### Data preprocessing

Balanced data sets are important for training classifier data^46^. The classifier only predicts most class data in the sample and completely ignores a few class data when the proportion of the majority of class data to total sample data is much larger than that of the minority class data^47^. In our experiment, Table 4 shows the proportion of rainfall and nonrainfall data for the two stations in different years, indicating that this proportion is relatively larger (from 1:29 to 1:58). Therefore, a method is required to solve this problem. The downsampling method was applied to balance the two types of data. This method can delete parts of the data in most samples or add some artificially generated or duplicated data to a few samples to solve the problem of remarkable imbalance of sample data^48^. This strategy is generally used to solve the problem of data imbalance in large data samples^19^. The specific processing of this method can be summarized as follows: (1) new nonrainfall data sets are randomly extracted from nonrainfall data sets, and the size of the new data sets is the same as that of the rainfall data sets; (2) the rainfall and new nonrainfall data sets are combined into training data sets, and the proportion of rainfall and new nonrainfall data sets is 1:1; (3) these combined training data sets are used as the training sample data for the BP–NN algorithm^19^.

**Table 4:** Statistical result of the ratio between rainfall data and nonrainfall data at the two stations.

Type	SNUS			NTUS		
	2010	2011	2012	2010	2011	2012
Rainfall data (epoch)	948	2469	2119	2099	2314	2216
Non-rainfall data (epoch)	54674	93025	84911	90486	67624	85073
Proportion	1:58	1:38	1:40	1:43	1:29	1:38

The weight became extremely large through the build up of accumulators due to the different dimensions and large numerical differences in different meteorological parameters. Moreover, the BP–NN algorithm is difficult to converge if the data are directly input into the model. Therefore, maximum and minimum methods were used to normalize the seven types of balanced data^23,42^. The balanced and normalized data were regarded as training data and input into the BP–NN model to establish the nonlinear relationship between the seven types of meteorological parameters and rainfall.

### Simulated experiment

The number of the input layer node was 7 for the BP–NN algorithm because of the number of input parameters (T, P, RH, PWV, MoH, HoD, and DoY). The number of the hidden layer node and the learning rate was calculated based on Eqs. (13) and (14). In this study, the values were 15 and 0.125, respectively. The number of the output layer node was 1 in the simulated experiment. Therefore, the structure of the BP–NN algorithm was 7–15–1. Sigmoid and ReLU functions were used for the transfer function of the hidden and output layers, respectively. The initial weight of the BP–NN model was generated based on the Nguyen–Widrow algorithm, and the BP–NN model was optimized by using the L–M optimal weight method.

The experiments focused on whether the rainfall occurred and not on the size of the rainfall. Therefore, the actual and simulated rainfall results were considered binary values. The actual rainfall was set to 0 when the rainfall was equal to 0 mm and 1 when the rainfall was larger than 0 mm. Negative values were observed in the simulated result because the simulated rainfall based on the BP–NN algorithm oscillated at approximately 0 mm when no rainfall occurred. Therefore, selecting an appropriate rainfall threshold was necessary to determine whether or not rainfall will occur. The specific method set a rainfall threshold (N). The simulated rainfall less than or equal to N mm was set to 0, whereas the simulated rainfall greater than N mm was set to 1.

The following indices were introduced to evaluate the result of the rainfall forecast model based on the improved BP–NN algorithm, namely, true (TFR), false (FFR), and missed (MFR) forecast rates:

15 $$\begin{array}{c}TFR=\frac{{N}_{true}}{{N}_{actual}}\\ FFR=\frac{{N}_{false}}{{N}_{actual}}\\ MFR=\frac{{N}_{missed}}{{N}_{actual}}\end{array}$$

where ${N}_{true}$ is the number of forecasted rainfall events of the model, ${N}_{actual}$ is the actual number of rainfall events, ${N}_{false}$ is the number of forecasted rainfall events but no rainfall actually occurred, and ${N}_{missed}$ is the number of forecasted rainfall events that the model failed to predict.

**Figure 5:**
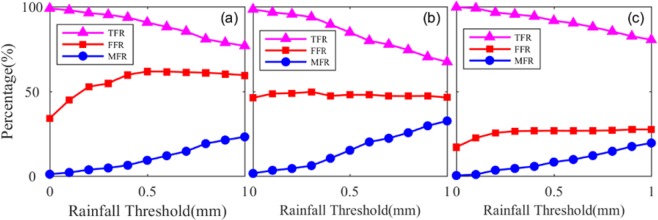
Simulated results of rainfall events for the three schemes at the SNUS station: Schemes (**a**) 1, (**b**) 2, and (**c**) 3. [the figure is plotted by MATLAB 2016a (https://cn.mathworks.com/products/matlab.html)].

Figure 5 shows the simulated result of Schemes 1, 2, and 3 in the SNUS station. This figure shows that TFR generally decreased and FFR and MFR increased with increasing rainfall threshold. The rainfall threshold with a value of 0 mm was the best among the simulated results of all schemes. Therefore, the rainfall threshold (N) of 0 mm was selected as the simulated rainfall result. Table 5 shows the statistical result of the simulated forecasting experiment of the three schemes in the two stations. In the table, the TFR of the simulated result of the three schemes is larger than 98%, whereas the FFR ranged from 17–47% in the two stations. In addition, this table indicates that the TFR of Schemes 2 and 3 was comparable, whereas the FFR decreased when more training data were used to establish the rainfall forecast model based on the improved BP–NN algorithm. The average values of TFR, FFR, and MFR of the three schemes in the two stations were 99.18%, 33.90%, and 0.82, respectively. These results validated the feasibility of the proposed rainfall forecast model based on the improved BP–NN algorithm.

**Table 5:** Statistical results of the simulated experiment of the three schemes at SNUS and NTUS stations.

Scheme	SNUS			NTUS		
	TFR/%	FFR/%	MFR/%	TFR/%	FFR/%	MFR/%
1	98.93	34.22	1.07	99.11	42.23	0.89
2	98.43	46.34	1.57	99.63	36.92	0.37
3	99.68	17.16	0.32	99.31	26.52	0.69

### Forecasted experiment

**Figure 6:**
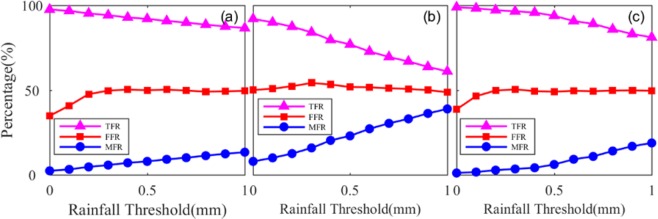
Forecasted results of rainfall events for the three schemes at the SNUS station: Schemes (**a**) 1, (**b**) 2, (**c**) 3. [the figure is plotted by MATLAB 2016a (https://cn.mathworks.com/products/matlab.html)].

**Figure 7:**
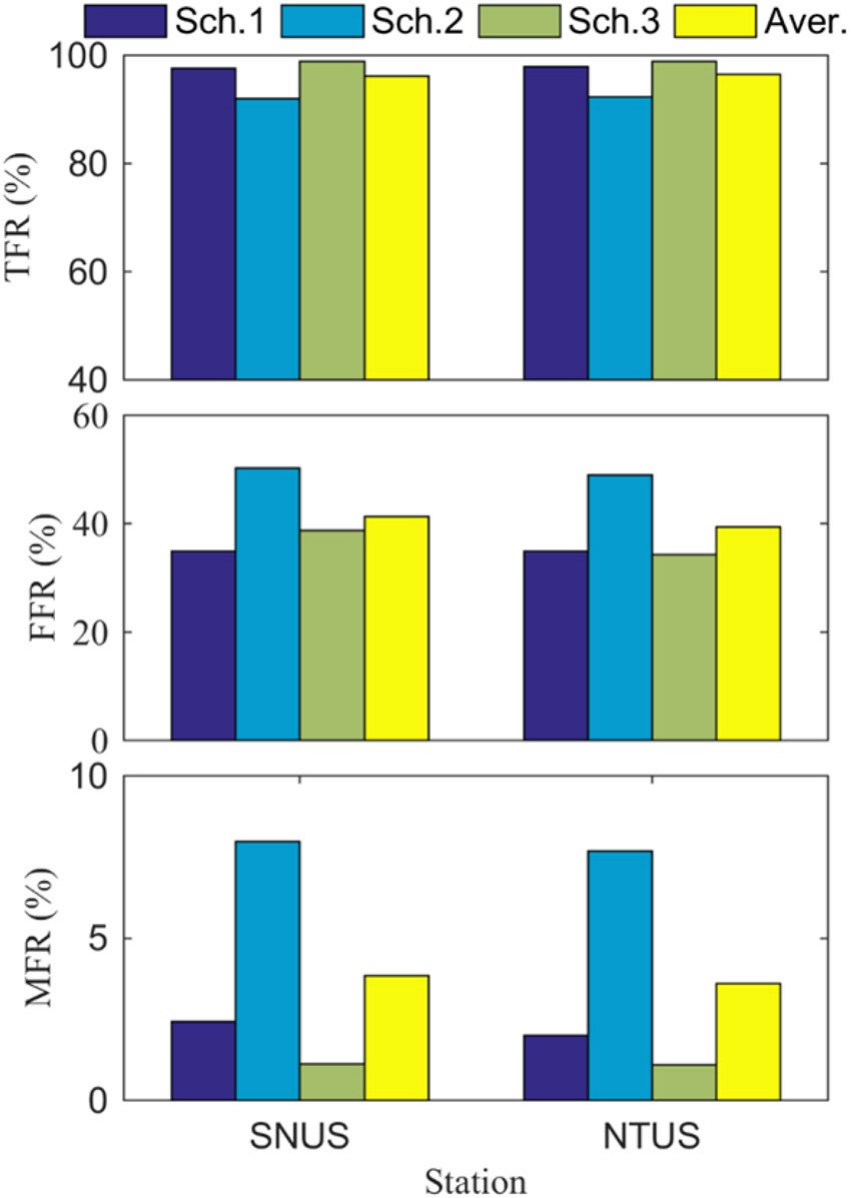
Forecasted results of the three schemes at SNUS and NTUS stations [the figure is plotted by MATLAB 2016a (https://cn.mathworks.com/products/matlab.html)].

In this section, the proposed rainfall forecast model was applied for rainfall forecasting in the two stations on the basis of the schemes designed in Table 6. The proposed model based on the BP–NN algorithm could forecast rainfall 10–60 minutes in advance. Figure 6 presents the forecasted rainfall result based on the BP–NN algorithm at the SNUS station, indicating that the best result could be obtained when the rainfall threshold was 0 mm. Therefore, this rainfall threshold was also determined in the forecasted experiment. This phenomenon also further verified the rationality of the strategy of selecting the rainfall threshold. Figure 7 shows the forecasted results of the three schemes in the two stations. The TFR and FFR of the proposed rainfall forecast model with the improved BP–NN algorithm could reach up to 92% to 99% and 35% to 43%, respectively. This figure also shows that the average TFR and of the three schemes are above 96% and approximately 40%, respectively. These results improved by approximately 10% with respect to TFR, and FFR is comparable to that of Manandhar *et al*.^19^.

**Table 6:** Statistical results of the forecasted experiment of the three schemes at the two stations for different levels of rainfall (0–50 mm/h; 0–100 mm/h and >100 mm/h).

Scheme	0–50 mm/h			50–100 mm/h			>100 mm/h		
	TFR%	FFR%	MFR%	TFR%	FFR%	MFR%	TFR%	FFR%	MFR%
1	97.92	13.07	2.08	100	13.60	0	100	19.53	0
2	92.76	23.21	7.24	98.28	13.24	0.72	99.84	8.77	1.16
3	99.00	5.67	1.01	100	1.38	0	100	5.33	0
Aver.	96.56	13.98	3.44	99.42	9.41	0.24	99.95	11.21	0.39

Table 6 shows the statistical forecasted result of the two stations for the three schemes under different levels of rainfall (0–50 mm/h; 0–100 mm/h and >100 mm/h). It can be concluded that the larger the rainfall, basically, the higher predictability the model has. In addition, the statistical result reveals that the averaged TFR, FFR, and MFR of the different schemes were 96.28%, 40.36%, and 3.72%, respectively. These results were superior to the forecasted result of previous studies that used GNSS-derived ZTD or PWV data^3,4,6,10^. In addition, it also can be observed from Table 6 that the forecasted result of Scheme 3 was superior to that of Scheme 2, especially under the case of rainfall <0–50 mm/h. Schemes 2 and 3 were designed to forecast rainfall in 2012 at the two stations by using different trained models. Two years of data (2010–2011) were used to train the rain forecast model for Scheme 3, whereas only one year of data (2011) was used for Scheme 2, further demonstrating that more trained data can improve the ability of describing the rainfall forecast model. Therefore, a better forecasted result could be obtained in Scheme 3. This result also indicated that the proposed rainfall forecast model should be trained by using as much data as possible.

## Conclusion

The correlation analysis between rainfall and different meteorological factors was performed. The results showed that no strong correlation existed between rainfall and any meteorological factor, thereby indicating that the occurrence of rainfall depends on a myriad of atmospheric parameters. Therefore, a rainfall forecast model based on the improved BP–NN algorithm was proposed by using multiple meteorological parameters. Two key parameters (the number of hidden layer nodes and learning rate) were determined based on the Kolmogrov’s theorem and empirical principle. The data on the two stations from 2010 to 2012 were used to train and validate the proposed BP–NN model. The simulated result of the BP–NN model in the two stations revealed the good performance of the proposed model with the average RFR and WRF of 99.18% and 33.90%, respectively. The forecasted result revealed that the rainfall could be forecasted 10–60 minutes in advance with the average RFR and WRF of 96.28% and 40.36%, respectively. These results verified the reliability and feasibility of the proposed rainfall forecast model based on the improved BP–NN algorithm. In addition, more data should be used to train the rainfall forecast model. In future studies, WFR should be decreased further by optimizing the selection of parameters in the BP–NN algorithm. Moreover, other rainfall forecast methods must be explored through different machine learning algorithms, such as SVM and long short-term memory, to improve the WFR of rainfall forecasting.
